# Targeting PKC as a Therapeutic Strategy to Overcome Chemoresistance in TNBC by Restoring Aurora Kinase B Expression

**DOI:** 10.1111/jcmm.70464

**Published:** 2025-03-18

**Authors:** Bing Cheng, Jinxin Chen, Vera Katalina, Guojie Long, Chaoying Wei, Zhitong Niu, Chen Chen, Panpan Wang, Qiang Yu, Wenyu Wang

**Affiliations:** ^1^ Guangdong Provincial Key Laboratory of Colorectal and Pelvic Floor Disease The Sixth Affiliated Hospital, Sun Yat‐Sen University Guangzhou China; ^2^ Guangdong Institute of Gastroenterology The Sixth Affiliated Hospital, Sun Yat‐Sen University Guangzhou China; ^3^ Biomedical Innovation Center The Sixth Affiliated Hospital, Sun Yat‐Sen University Guangzhou China; ^4^ Genome Institute of Singapore, Agency for Science, Technology, and Research (A*STAR) Singapore; ^5^ Department of General Surgery (Department of Pancreatic Hepatobiliary Surgery) The Sixth Affiliated Hospital, Sun Yat‐Sen University Guangzhou China; ^6^ The First Affiliated Hospital of Jinan University Guangzhou China; ^7^ Department of Physiology Yong Loo Lin School of Medicine, National University of Singapore Singapore Singapore; ^8^ Cancer and Stem Cell Biology DUKE‐NUS Medical School Singapore Singapore; ^9^ Tianfu Jincheng Laboratory Chengdu China

**Keywords:** AURKB, chemoresistance, GCN2‐p‐eIF2ɑ axis, PKC, TNBC

## Abstract

Triple‐negative breast cancer (TNBC) poses a significant challenge due to its high mortality rates, primarily attributed to resistance against chemotherapy regimens containing taxanes like paclitaxel. Thus, developing combinatorial strategies to override resistance is a pressing need. By taking advantage of a library screening with various kinase inhibitors, we found that the small‐molecule inhibitor enzastaurin targeting protein kinase C (PKC) could overcome resistance in TNBC cells. Mechanistically, dual treatment with paclitaxel and enzastaurin resulted in efficient mitotic arrest and subsequent cell death by restoring AURKB expression. Further analysis revealed that the GCN2‐p‐eIF2α axis was responsible for the posttranscriptional accumulation of AURKB upon combinatorial treatment. Finally, we confirmed that combinatorial regimens synergistically suppressed tumour growth in vivo in mouse models. Moreover, the efficiency of dual treatment was largely determined by AURKB, implying that AURKB could be a potential predictive marker for stratifying patients who may benefit from the combinatorial treatment. Collectively, our study not only unravels a novel underlying mechanism for paclitaxel resistance in TNBC but also provides a new potential combinatorial therapeutic strategy in the clinic.

## 1 Introduction

Breast cancer is the most common cancer and the second leading cause of death among women worldwide [1]. It is considered a heterogeneous disease with substantial morphological, pathological and genetic variation. It can be classified into four major subtypes depending on the expression of hormone receptors (progesterone receptor and oestrogen receptor) and human epidermal growth factor receptor (HER2). Triple‐negative breast cancer (TNBC) is the most aggressive subtype, accounting for approximately 15%–20% of all breast cancer patients. Due to the lack of expression of all three receptors, chemotherapy, which includes taxanes like paclitaxel, anthracyclines or a combination of both, remains one of the mainstays for treating TNBC patients [1, 2].

Although many TNBC patients initially respond well to chemotherapy containing paclitaxel, up to 60%–70% of patients will eventually undergo recurrence within 3–5 years with poor survival rates due to progressively acquired resistance. To explore the underlying mechanisms of resistance, most previous studies have focused on alterations in drug targets, such as tubulin mutations, or drug pharmacokinetics, and dysfunction of apoptotic signalling pathways. However, these studies have not yet been successfully translated into clinical use [3, 4]. Hence, a better understanding of the mechanisms leading to chemoresistance and the identification of novel strategies is of great importance. Alteration or reprogramming of the human kinome has played a vital role in regulating tumour progression, including cell proliferation, survival, migration and metastasis. Consequently, targeting protein kinases has been considered one of the most attractive therapeutic strategies in cancer treatment.

In this study, we have generated paclitaxel‐resistant TNBC cell lines from SUM159PT and MDA‐MB231 to mimic acquired chemoresistance in vitro. Based on a drug library screening with 180 protein kinase inhibitors, we have identified an inhibitor of the protein kinase C (PKC) family, enzastaurin, which resensitises resistant TNBC cells to paclitaxel. Enzastaurin has shown promising results in phase III clinical trials for the combination treatment of diffuse large B‐cell lymphoma patients and has been granted fast‐track designation by the FDA in 2020 for the treatment of Glioblastoma Multiforme [5, 6, 7]. Further experiments demonstrated that targeting PKC can restore the expression of Aurora Kinase B (AURKB) posttranscriptionally via the GCN2‐p‐eIF2α axis, ultimately restoring mitotic arrest and subsequent cell death in resistant TNBC cells. Moreover, pharmaceutical or genetic inhibition of AURKB could reverse the effect of sensitisation by enzastaurin both in vitro and in vivo. Collectively, our study proposes a new therapeutic approach to treat chemoresistant TNBC patients through cotargeting PKC together with paclitaxel treatment. AURKB expression may serve as a potent biomarker to allow stratification of patients potentially benefiting from this novel combinatorial regimen.

## 2 Materials and Methods

### 2.1 Cell Culture

MDA231‐P/R were cultured in DMEM supplemented with 10% FBS and 1% P/S. SUM159‐P/R were cultured in Ham's F‐12 Nutrient Mix medium and supplemented with 5% FBS, 10 mM HEPES, 5 ng/mL Insulin, 1 μg/mL hydrocortisone and 1% P/S.

### 2.2 Drug Screening

A compound library containing 180 small molecules targeting kinases was used for the screening. After 4 days of treatment, cell viability was measured using a Cell Titre‐Glo Luminescent Assay kit (Promega; G7570). *Z*‐score value was calculated by the formula: Z=$\frac{x-u}{\delta }$, where *x* stands for *H*‐score value, *μ* stands for mean and *σ* stands for standard deviation.

### 2.3 Lipid ROS Assay

SUM159‐P/R cells were treated with RSL3 or not for 24 h, followed by incubation with C11‐BODIPY‐containing medium for 30 min and subsequently subjected to flow cytometry analysis of lipid ROS levels.

### 2.4 Animal Experiments

Six to 8‐week‐old female BALB/c nude mice were purchased from Guangdong Gempharmatech Co. Ltd., China. SUM159‐PR‐shCtrl and shAURKB cells (2 × 10^6^) were subcutaneously injected on both flanks of mice. Once the tumour volume reached 100 mm^3^ on average, mice were treated with vehicle, Enza (10 mg/kg, i.p. daily), PTX (20 mg/kg, i.p., thrice/week) or in combination. Tumour volume was measured every 3 days using the following formula: tumour volume(mm^3^) = (length × width^2^) × 0.5.

### 2.5 IHC Staining

Tissue section slides were deparaffinised, rehydrated and antigens were retrieved using microwave. Samples were then stained with indicated primary antibodies overnight at 4°C. The IHC score was quantified as described previously.

### 2.6 Reagents

Detailed information on antibodies, chemicals, primers and the sequences of siRNA or shRNA is shown in Tables S1–S4.

Additional materials and methods are provided in the file named ‘Supporting Information’.

## 3 Results

### 3.1 Generation of Paclitaxel‐Resistant TNBC Cell Lines

**FIGURE 1:**
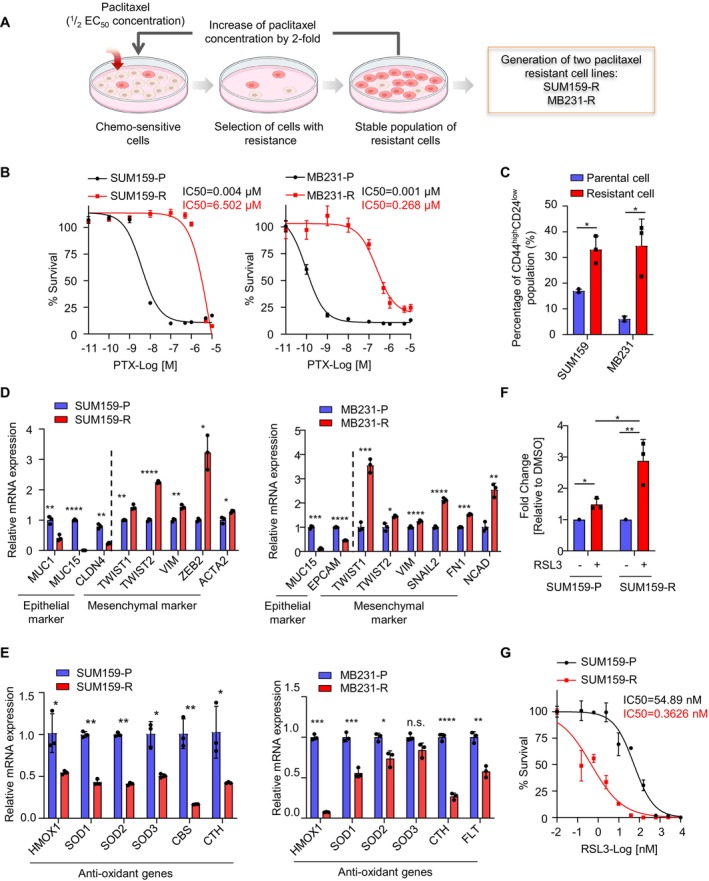
Paclitaxel‐resistant TNBC cells display features reminiscent of drug‐tolerant persister (DTP) cells. A. Graphical overview of Paclitaxel (PTX)‐resistant TNBC cell generation. B. Cell viability was measured 4 days after exposure to PTX in a dose titration manner. The IC_50_ of PTX for the indicated TNBC cell lines (P, parental cells; R, paclitaxel resistant) was calculated and shown on the graph. C. FACS analysis of the percentage of CD44^high^CD24^low^ breast cancer stem cells in both parental and resistant TNBC cells from three independent experiments. D and E. qRT‐PCR analysis of mRNA expression of indicated EMT markers and antioxidant genes in SUM159‐P, SUM159‐R, MB231‐P and MB231‐R cells. F. Analysis of lipid ROS levels in SUM159‐P and ‐R cells treated with RSL3 (1 μM) or not for 24 h. G. Measurement of the IC_50_ of RSL3 in SUM159‐P and ‐R cells. Data are presented as mean ± SEM. Statistics were acquired by two‐tailed unpaired Student's *t* test in C, D, E and F. *p < 0.05; **p < 0.01; ***p < 0.001; ****p < 0.0001; n.s.: Not significant.

To obtain paclitaxel‐resistant cell lines, parental TNBC cell lines including SUM159PT (hereafter referred to as SUM159‐P) and MDA‐MB231 (hereafter referred to as MB231‐P) were exposed to stepwise increasing concentrations of paclitaxel (PTX) repetitively, starting with half of the IC_50_ concentration. After months of selection, we generated resistant derivatives for each cell line stably growing in paclitaxel, which were termed SUM159‐R and MB231‐R. As expected, SUM159‐R and MB231‐R showed an approximate 2000‐ and 300‐fold increase in IC_50_ of PTX compared to the parental cell lines, respectively (Figure 1A,B). Accumulating evidence has pinpointed tumour‐initiating cells (TICs) to be the cause of chemoresistance in a variety of cancers due to their ability of self‐renewal and regenerating heterogeneity. Breast TICs are characterised by a high expression of CD44 and a low or absent expression of CD24 [8, 9]. Indeed, we found a significant increase in the TIC population in paclitaxel‐resistant cell lines determined by CD44 high and CD24 low expression, indicating enrichment of TICs during the acquisition of chemoresistance (Figure 1C).

Emerging evidence implicates that drug‐tolerant persister (DTP) cells represent a reservoir of residual surviving cells, which are the chief culprits responsible for therapy failure and tumour relapse [10, 11, 12]. To explore whether the resistant cells generated in this study also possess features resembling DTP cells, we analysed key characteristics of DTP, including aspects of epithelial‐to‐mesenchymal transition (EMT), reduced antioxidant ability and increased sensitivity to ferroptosis in SUM159‐R and MB231‐R cells [13, 14, 15]. Intriguingly, we observed a drastic decrease in epithelial markers for breast cancer cells such as CLDN4, MUC1, MUC15 or EPCAM, simultaneously accompanied by a notable increase in mesenchymal cell markers including TWIST1/2, VIM, ZEB2, ACTA2, SNAIL2, FN1 and NCAD, supporting EMT induction in the resistant cells compared to their parental counterparts. Furthermore, consistent with disabled antioxidant capabilities in DTP, we found that the resistant cells showed markedly reduced expression of several antioxidant genes including HMOX1, SOD1/2/3, CBS, CTH and FLT, indicating an impaired ability of resistant cells to counteract oxidant stress [16, 17, 18] (Figure 1D,E). In accordance, reactive oxygen species (ROS)‐induced lipid peroxidation was analysed using the fluorescence probe C11‐BODIPY. As expected, the resistant cells showed a significant increase in ROS levels upon exposure to the GPX4 inhibitor, RSL3, which is a typical inducer of ferroptosis, an oxidative form of cell death. Consistently, we also found a relatively high sensitivity of resistant cells to GPX4 inhibition demonstrated by the dramatic decrease in IC_50_ of RSL3 in SUM159‐R cells compared to the parental cells (Figure 1F,G). Taken together, these analyses suggested the successful establishment of an in vitro PTX‐resistant TNBC cell model that displays key features of DTP, thus giving rise to drug resistance.

### 3.2 A Kinase Inhibitor Screen Identified Compounds That Sensitise Paclitaxel‐Resistant Cell Lines

**FIGURE 2:**
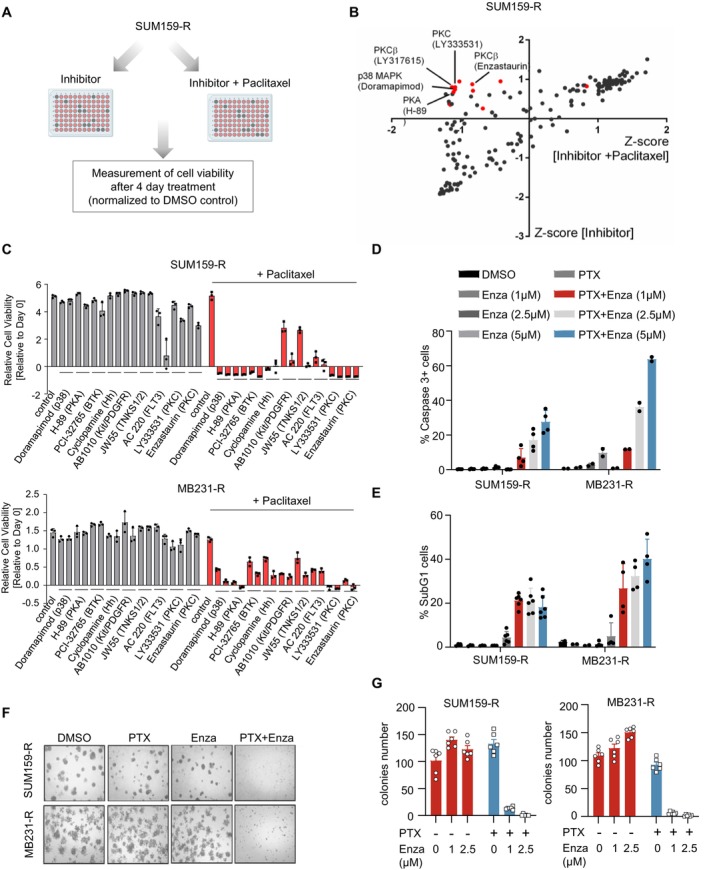
A kinase inhibitor library‐based screening identifies compounds that sensitise‐resistant TNBC cells to paclitaxel. A. Schematic depiction of the drug library screening. B. The scatter plot shows the *Z*‐scores of the single treatment vs. the combination treatment in SUM159‐R cells. The dots in the II quadrant represent compounds that can significantly reduce cell viability in combination with PTX while having minimal effect as a single treatment. C. Measurement of cell viability 4 days after treatment with single candidate kinase inhibitors at doses of 1 and 5 μM or in combination with PTX respectively, in both SUM159‐R and MB231‐R cells. D, E. FACS analysis of the percentage of apoptotic/dead cells upon single treatment or in combination in both SUM159‐R and MB231‐R cells. F, G. Tumour sphere formation and anchorage‐independent colony formation assay in both SUM159‐R and MB231‐R cells.

To explore novel strategies potentially able to override chemotherapeutic resistance, we performed drug screening consisting of 180 small‐molecule inhibitors targeting various kinases based on our established cell model. To do so, SUM159‐R was treated with either the inhibitor alone or in combination with PTX (Figure 2A). After 4 days of treatment, cell viability was measured and the *Z*‐score was calculated for each single and combination treatment. All measurements of either single or combination treatment were taken as one population and the *Z*‐score calculates the distance of each measurement from the population mean. We then plotted the *Z*‐scores of the single treatment versus the combination treatment on a scatter plot, whereby the data points in the II quadrant represent those compounds which have minimal effect as single treatments but can significantly reduce cell viability in combination with PTX and are most interesting to us (Figure 2B). Notably, three of the inhibitors in this quadrant target the protein kinase C family (enzastaurin/LY317615 and LY333531), in addition to others, such as inhibitors targeting p38 MAPK (doramapimod), protein kinase A (H‐89), Bruton's tyrosine kinase (BTK) and the hedgehog pathway (cyclopamine), which have previously been found to play important roles in chemoresistance among broad cancers including colorectal, breast and prostate cancer [19, 20, 21, 22]. Protein kinase C (PKC) is a family of serine/threonine kinases that regulate signalling pathways associated with proliferation, invasion and metastasis and have been implicated in the progression of various cancer entities, including breast cancer, while its role in mediating chemoresistance remains largely unknown [23, 24]. Additionally, enzastaurin (Enza) is an orally administered drug that was intended for the treatment of various cancers and has since entered clinical trials [5, 6, 7]. Hence, for further validation, we focus on small‐molecule inhibitors targeting PKC. We validated the two PKC inhibitors together with seven other candidates including the aforementioned four published targets and three novel targets in chemoresistance, the Kit/Platelet‐derived growth factor receptor (PDGFR), tankyrase 1/2 (TNKS1/2) and Fms‐related tyrosine kinase 3 (FLT3) at 1 and 5 μM [25, 26, 27], and measured cell viability after 4 days of treatment. All inhibitors reduced cell viability in combination with PTX but had minimal effect as a single treatment (Figure 2C). Moreover, the inhibitors had no significant effect on cell viability in the parental cell lines (Figure S1). To further determine the exact effect of Enza treatment in sensitising cells, we measured apoptosis and cell death by active caspase3 or PI staining in resistant cells. The results demonstrated that Enza alone had little effect on apoptosis, whereas the combination with PTX led to a dose‐dependent increase in the number of apoptotic cells (Figure 2D). This was further confirmed by measuring the subG1 fraction which correlates with the amounts of cells undergoing cell death based on DNA fragmentation (Figure 2E). As mentioned above, chemoresistant cells generally display high stemness properties. To explore whether the combination could largely suppress the stemness of TNBC cells, we performed the 3D tumoursphere and anchorage‐independent colony formation assays. As shown, combination treatment dramatically inhibited the formation of tumourspheres and reduced colony numbers, while single treatment had no such effect (Figure 2F,G). Collectively, these results suggested that combinatorial treatment with Enza was able to reverse PTX resistance in TNBC cell lines.

### 3.3 Enzastaurin Reinduced Mitotic Arrest in Resistant TNBC Cells Upon Combination With Paclitaxel by Targeting PKCα/δ

In addition to PTX, microtubule‐disrupting agents like vincristine (VCR) and anthracycline antibiotics like adriamycin (ADR) are the other two types of commonly used chemotherapeutic compounds [28, 29]. To test whether sensitisation with Enza is selective for microtubule‐targeting agents, we first determined the sensitivity of parental and resistant cell lines to VCR or ADR in the presence or absence of Enza. In contrast to VCR, ADR does not interfere with microtubules but rather inhibits biosynthesis by intercalating with the DNA and stopping the progression of topoisomerase II. Interestingly, we found that both SUM159‐R and MB231‐R also showed resistance to VCR and ADR compared to their parental counterparts. However, treatment with Enza could significantly increase sensitivity to VCR, as measured by the IC_50_, but to a much lesser extent to ADR, especially in MB231‐R cells (Figure S2A,B). Additionally, dual treatment with Enza and VCR also significantly enhanced apoptosis, whereas it showed little effect together with ADR (Figure S2C,D). These results implied that the resensitisation by Enza was restricted to microtubule‐disrupting compounds, whether they acted by stabilising or destabilising microtubules.

**FIGURE 3:**
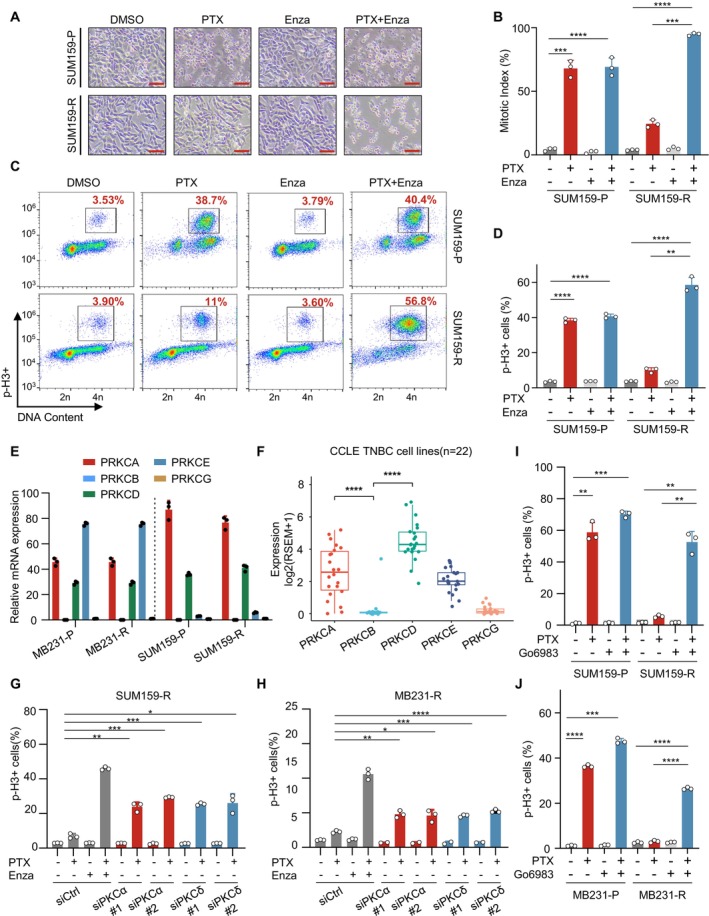
Combinatorial treatment with enzastaurin induces mitotic arrest in resistant TNBC cells upon treatment with paclitaxel by targeting PKCα/δ. A and B. Representative images showing round‐up cells arrested in mitosis upon single or combination treatment in SUM159‐P and SUM159‐R cells. Scale bar: 100 μM. The plot shows statistical analysis of the Mitotic Index defined by the percentage of mitotic cells among total cells upon indicated treatment from three independent experiments. C, D. FACS analysis of mitotic cells costained with phosphorylated histone 3 (p‐H3, mitotic marker) and PI (DNA content) upon indicated treatment in SUM159‐P and SUM159‐R cells. Representative images showing the proportion of mitotic cells upon indicated treatment as shown in (C). Statistical analysis of three independent experiments as shown in (D). E. qRT‐PCR analysis of various PKC isoforms potentially targeted by Enza in both parental and resistant TNBC cells (PRKCA, PRKCB, PRKCD, PRKCE and PRKCG encoding PKCα, PKCβ, PKCδ, PKCε and PKCγ, respectively). F. Analysis of mRNA expression of the indicated PKC isoforms mentioned in (E) based on the CCLE‐BRCA database (*n* = 22 TNBC cell lines). G, H. FACS analysis of mitotic cells costained with p‐H3 and PI following transfection with control siRNA or two independent siRNAs targeting PKCα or PKCδ upon indicated treatment in SUM159‐R cells (G) and MB231‐R cells (H). The plot shows statistical analysis from three independent experiments. I, J FACS analysis of mitotic cells costained with p‐H3 and PI upon treatment with either PTX/PKC inhibitor (Go6983) alone or in combination in SUM159‐P&R cells (I) and MB231‐P & R cells (J). The plot shows statistical analysis from three independent experiments. Data are presented as mean ± SEM. Statistics were acquired by two‐tailed unpaired Student's *t* test in B, D, F, G, H, I and J. *p < 0.05; **p < 0.01; ***p < 0.001; ****p < 0.0001; n.s.: Not significant.

Microtubule poisons such as paclitaxel exert their function by perturbation of microtubule dynamics, thereby forcing cells to arrest in mitosis. Depending on the cell type and biological context, cells blocked in mitosis can undergo cell death or alternatively prematurely escape mitosis termed ‘slippage’ [3, 30, 31]. To study how Enza may influence mitosis, we first tracked morphological changes of both parental and resistant TNBC cell lines treated with PTX/Enza alone or in combination under microscopy. Not surprisingly, we observed that the combinatorial treatment was capable of inducing mitotic arrest, similar to levels in the parental counterpart treated with PTX alone (Figure 3A,B). Furthermore, we performed FACS analysis of the mitotic marker, phosphorylated histone 3 (p‐H3), together with DNA content labelling by propidium iodide (PI). Combinatorial treatment with Enza successfully restored mitotic arrest in both resistant cell lines which was largely suppressed upon PTX treatment alone (Figures 3C,D and S3). Given that Enza was initially designed as a potent inhibitor targeting PKCβ but also displayed pan‐PKC inhibition activity in the cells [32], we next sought to determine the Enza‐targeted isoform(s) in our context. To this end, we first examined mRNA levels of diverse PKC isoforms that could be potentially targeted by Enza [33, 34]. Intriguingly, PRKCA and PRKCD rather than PRKCB showed relatively higher expression in both SUM159 and MB231 parental and resistant cells (Figure 3E). Next, to determine whether this phenomenon is common across TNBC cells, we referred to CCLE datasets and analysed mRNA expression of diverse PKC isoforms in 22 human TNBC cell lines. In line with the aforementioned result, we found that the mRNAs of PRKCA and PRKCD were highly expressed compared to PRKCB (Figure 3F). Hence, to determine whether PKCα or PKCδ(encoded by PRKCA or PRKCD respectively) is the main target of Enza under combinatorial treatment in resistant cells, we delivered two independent siRNAs targeting PKCα or PKCδ into SUM159‐R and MB231‐R cells followed by PTX treatment and measured the proportion of mitotic cells by FACS. Silencing of PKCα or PKCδ was able to restore mitotic arrest upon treatment with PTX in both resistant cells which largely mimicked the effect of Enza (Figures 3G,H and S4A,B). Additionally, we calculated the mitotic index which is determined by measuring the percentage of round‐up mitotic cells among total cells in the same field upon PTX alone or in combination treatment. Similar to the aforementioned result, in the absence of PKCα/δ, PTX alone efficiently induced mitotic arrest resembling the effect of combination with Enza in both SUM159‐R and MB231‐R cells (Figure S4C–F). Moreover, Go‐6983, a generally used PKC inhibitor, was further introduced to potently target PKCα/δ. We found that pharmaceutical inhibition of PKCα/δ in combination with PTX also drastically rescued mitotic arrest in both resistant cells (Figures 3I,J and S5) [34, 35]. Taken together, these data suggested that combinatorial treatment with Enza restored mitotic arrest in resistant cells via targeting PKCα/δ.

### 3.4 Resensitisation of Resistant TNBC Cells to Paclitaxel by Enzastaurin Relies on Aurora Kinase B Accumulation

**FIGURE 4:**
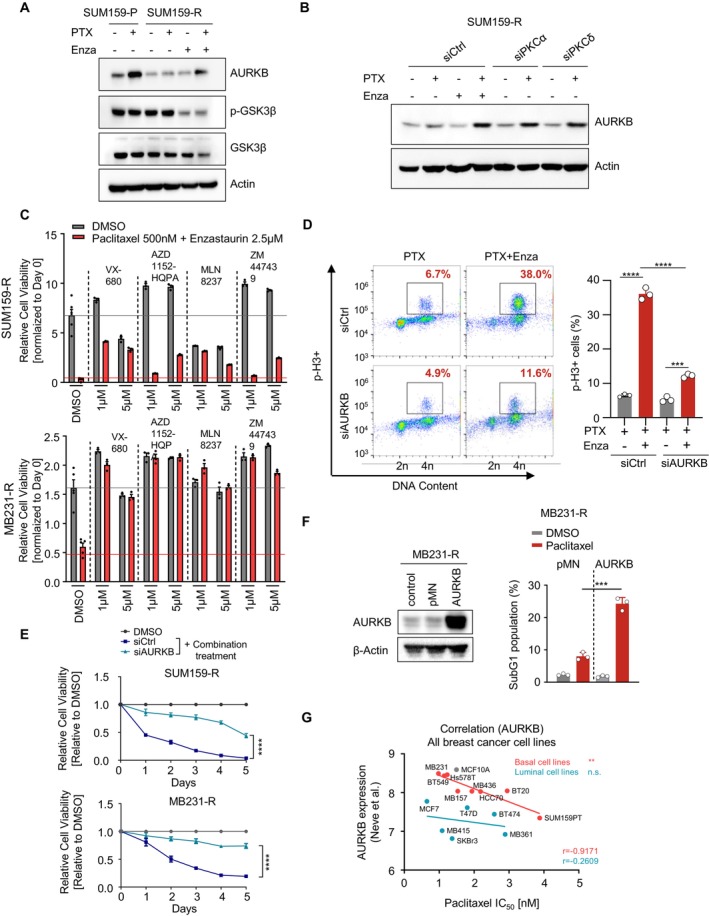
Resensitisation of resistant TNBC cells by enzastaurin depends on AURKB accumulation. A. Immunoblotting analysis of indicated markers upon treatment with PTX/Enza alone or in combination in SUM159‐P and ‐R cells. B. Immunoblotting analysis of AURKB following transfection with siRNA targeting PKCα, PKCδ or control siRNA under indicated treatment in SUM159‐R cells. C. The graph shows cell viability of both SUM159‐R and MB231‐R cells after PTX treatment alone or in combination with Enza in the presence or absence of various AURKB inhibitors. D. FACS analysis of mitotic cells with or without AURKB silencing upon PTX alone or combinatorial treatment with Enza in SUM159‐R cells. E. The plot shows the cell viability of both SUM159‐R and MB231‐R cells upon exposure to combinatorial treatment with or without siRNA targeting AURKB at the indicated time points. F. Left: Immunoblotting shows ectopic AURKB expression in MB231‐R cells. Right: The plot shows the percentage of dead cells upon indicated treatment measured by FACS analysis. G. The plot shows a correlation between sensitivity to PTX determined by measurement of IC_50_ and AURKB expression level in a panel of basal‐like and luminal breast cancer cell lines. (Red and blue dots represent basal‐like and luminal breast cancer cell lines, respectively.) Data are presented as mean ± SEM. Statistics were acquired by two‐tailed unpaired Student's *t* test in D, E, F and G. **p < 0.01; ***p < 0.001; ****p < 0.0001; n.s.: Not significant.

Given that aurora kinase B, a central component of the chromosomal passenger complex (CPC), is required for proper mitosis and modulates the response to taxane‐based chemotherapy [36, 37], we next sought to test the role of Enza in the regulation of AURKB. First, we examined AURKB expression in both parental and resistant cells under PTX alone or in combination conditions. As anticipated, PTX was able to efficiently induce AURKB expression in parental cells. Conversely, AURKB accumulation induced by PTX was remarkably suppressed in resistant cells. Intriguingly, combinatorial treatment with PTX and Enza significantly restored AURKB expression. The phosphorylation of GSK3β on serine 9, a marker indicating the activity of PKC [38], substantially decreased upon exposure to Enza, suggesting efficient inhibition of PKC by Enza (Figure 4A). Consistently, silencing of PKCα/δ by siRNAs also resembled the effect of dual treatment in restoring AURKB expression, further supporting that PKCα and PKCδ were the primary targets of Enza in this context (Figure 4B). To determine the role of AURKB in regulating the response to combinatorial treatment, we first utilised several inhibitors, including VX‐680, AZD1152‐HQPA, MLN8237 and ZM447439 to block AURKB activity. The data demonstrated that pharmaceutical inhibition of AURKB dramatically blunted the resensitisation effect of Enza in the resistant cell lines [39, 40] (Figure 4C). We further delivered control siRNA or siRNA targeting AURKB into resistant cell lines and assessed mitotic arrest by FACS following PTX treatment alone or in combination with Enza. In line with the aforementioned results, direct silencing of AURKB could profoundly impede mitotic arrest induced by combinatorial treatment, indicating the essential role of AURKB in regulating mitotic arrest upon exposure to dual compounds (Figure 4D). Accordingly, the resensitisation effect of Enza was largely impaired by the silencing of AURKB (Figure 4E). On the contrary, ectopic expression of AURKB could resensitise resistant cells to PTX (Figure 4F). We further measured the IC_50_ of PTX in a panel of basal‐like and luminal breast cancer cell lines, for which AURKB mRNA expression levels were obtained from the dataset on Gene Expression‐Based Outcome for Breast Cancer Online (GOBO) [41]. Specifically, we found a significant inverse correlation between AURKB expression and IC_50_ of PTX in basal‐like subtype, the majority of which belong to TNBC (*r* = −0.9171, *p* = 0.0013), but not luminal breast cancer cell lines (*r* = −0.2609, *p* = 0.6175) (Figure 4G). These data suggested a positive correlation between AURKB expression and PTX sensitivity in TNBC cells. Therefore, revitalising AURKB expression such as by targeting PKC could be an important strategy to override PTX resistance in TNBC. Taken together, these data highlighted the importance of AURKB as a determinant in mediating sensitisation to PTX and the newly identified combinatorial treatment in resistant TNBC cell lines.

### 3.5 Paclitaxel and Enzastaurin in Combination Regulate Aurora Kinase B Expression Posttranscriptionally Through the GCN2 ‐p‐eIF2α Axis

**FIGURE 5:**
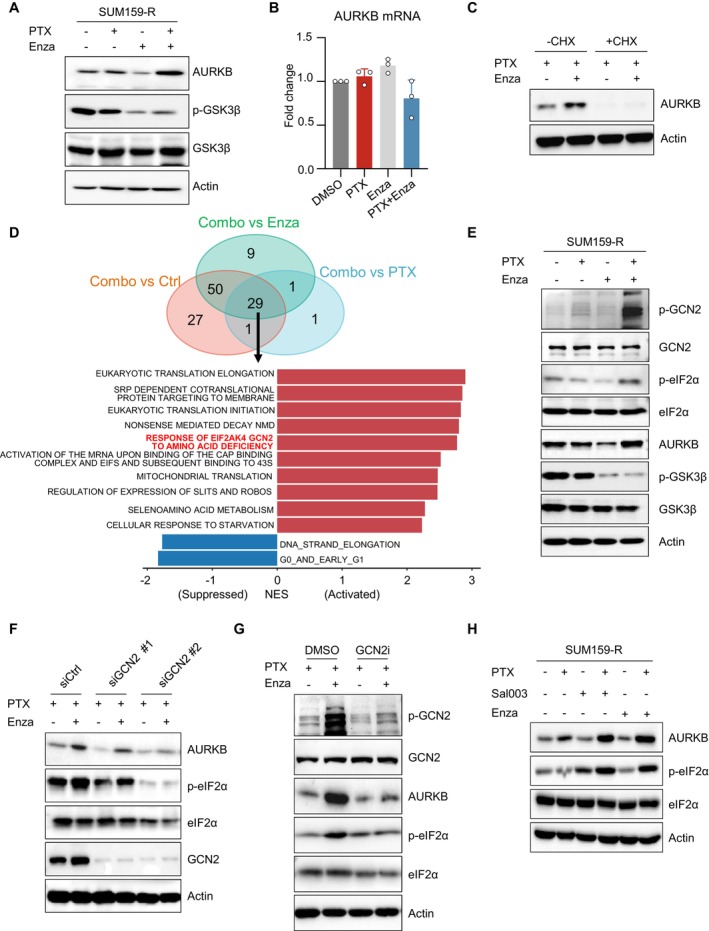
Enzastaurin restores AURKB expression in paclitaxel‐resistant TNBC cells in a posttranscriptional manner depending on the GCN2‐p‐eIF2α axis. A, B. Immunoblotting and qRT‐PCR analysis of AURKB expression upon PTX/Enza single treatment or in combination in SUM159‐R cells. C. Immunoblotting analysis of AURKB in the presence or absence of cycloheximide (CHX) upon PTX alone or in combination treatment in SUM159‐R cells. D. RNA‐seq analysis of SUM159‐R cells upon PTX/Enza single treatment or combination treatment. Top: Venn diagram shows the number of enriched signalling pathways in the combination treatment group compared to the other three groups analysed by GSEA. Bottom: Histogram shows the top 10 positively enriched (red columns) and the two negatively enriched signalling pathways (blue columns). (NES indicates normalised enrichment score, *p* < 0.05). E. Immunoblotting analysis of the indicated markers involved in the GCN2‐p‐eIF2α axis in SUM159‐R cells. F. Immunoblotting analysis of indicated markers in the presence or absence of two individual siRNAs targeting GCN2 upon exposure to PTX alone or in combination in SUM159‐R cells. G. Immunoblotting analysis of indicated markers in the presence or absence of GCN2 inhibitor (GCN2iB, 1 μM) in SUM159‐R cells. H. Immunoblotting analysis of indicated markers upon treatment with PTX, Enza or the eIF2α phosphatase inhibitor (sal003, 10 μM) alone or in combination in SUM159‐R cells.

To decipher how combinatorial treatment would lead to increased expression of AURKB, we first examined AURKB expression at both the protein and mRNA levels upon single compound treatment or in combination. As expected, we observed evident accumulation of AURKB protein upon the combinatorial treatment, while there was not much change in AURKB mRNA (Figure 5A,B), implying that the combinatorial treatment promoted AURKB accumulation via a posttranscriptional mechanism. Since rapid protein degradation mediated by the ubiquitin‐proteasome system was responsible for the refined control of AURKB expression during mitosis [42, 43], we next carried out a cycloheximide chase experiment to determine whether combinatorial treatment could enhance protein stability of AURKB, by which cycloheximide (to inhibit de novo protein synthesis) was added 24 h after exposure to PTX or in combination with Enza. In comparison with PTX alone, dual compound treatment modestly increased the half‐life of AURKB, indicating other potential mechanisms resulting in robust AURKB accumulation (Figure S6A,B). Interestingly, when we pretreated the cells with cycloheximide 6 h before combinatorial treatment, the accumulation of AURKB was almost completely blocked (Figure 5C). This suggested that the accumulation of AURKB upon combinatorial treatment was largely due to de novo protein synthesis.

To explore the underlying mechanisms accounting for AURKB upregulation, we performed RNA‐seq analysis in SUM159‐R cells with the vehicle, single or combinational treatment. Gene set enrichment analysis (GSEA) was performed, and 29 shared signalling pathways were found to be enriched in combinatorial treatment compared to other groups in resistant cells. Among them, GCN2‐associated signalling was highly enriched in the combination group (Figure 5D). GCN2 was a kinase that phosphorylated eIF2α at serine 51 (p‐eIF2α) in response to various stress stimuli, including amino acid deficiency, ER stress and DNA damage. The activation of the GCN2‐eIF2α pathway, leading to reduced global protein synthesis and simultaneously coupled with increased translation of selective mRNAs, assisted cells in counteracting the harsh conditions [44, 45, 46]. We speculated that the GCN2‐eIF2α axis might play a role in promoting AURKB expression posttranscriptionally in resistant cells. To validate this, we first examined the activation of the GCN2‐eIF2α axis in resistant cells under indicated treatments (Figure 5E). Indeed, combinatorial treatment dramatically induced GCN2 phosphorylation along with its downstream phosphorylation of eIF2α, simultaneously accompanied by AURKB accumulation (Figure 5E). Next, we asked whether depletion of GCN2 could reverse the effect of combinatorial treatment on AURKB and p‐eIF2α. As expected, two independent siRNAs targeting GCN2 were able to diminish the phosphorylation of eIF2α and accumulation of AURKB by combinatorial treatment (Figure 5F). Not surprisingly, GCN2iB, an ATP‐competitive inhibitor of GCN2, also showed a similar effect as GCN2 silencing (Figure 5G). Additionally, to further confirm the role of p‐eIF2α in this context, sal003, a potent inhibitor blocking the activity of the eIF2α phosphatase Gadd34/PP1 and consequently increasing the phosphorylation of eIF2α [47, 48], was tested under indicated treatments. As shown, sal003 was capable of inducing effects similar to those of Enza in the resistant cells upon PTX treatment, which remarkably restored AURKB accumulation along with increased levels of phosphorylated eIF2α (Figure 5H). Together, these results demonstrated that dual treatment with PTX and Enza mediated AURKB accumulation posttranscriptionally via the GCN2‐p‐eIF2α axis in resistant cells.

### 3.6 Aurora Kinase B Determines the Synergistic Effect of Paclitaxel and Enzastaurin In Vivo

**FIGURE 6:**
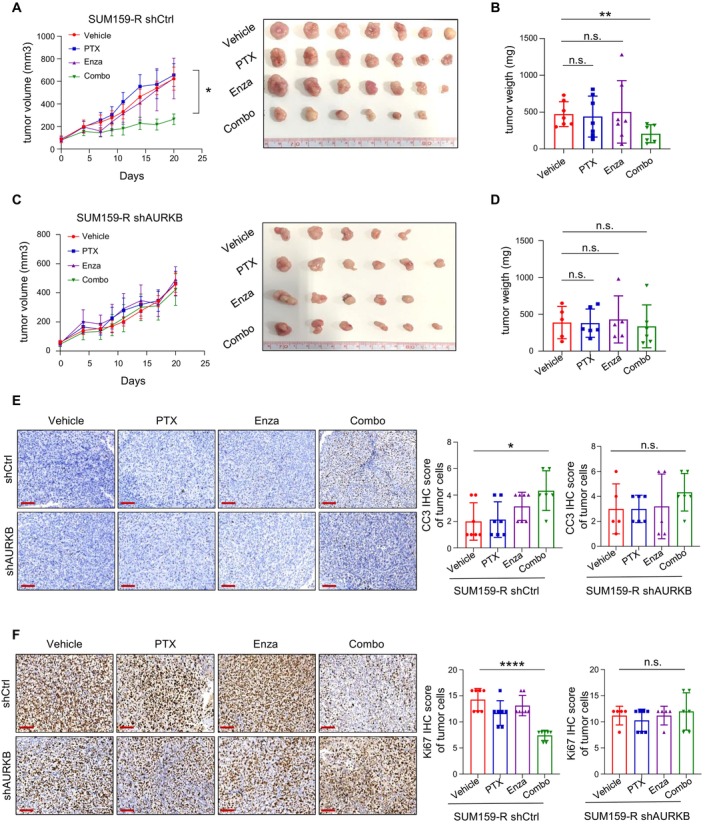
The synergistic inhibitory effect of paclitaxel and enzastaurin depends on AURKB expression. A–D. Tumour growth and weight of SUM159‐R xenografts with control shRNA (shCtrl) or shRNA targeting AURKB (shAURKB) upon treatment with PTX/Enza alone or in combination. E, F. IHC staining of cleaved caspase3 (CC3) and Ki67 in the tumour sections from the shCtrl or shAURKB group upon PTX/Enza alone or combinatorial treatment. Scale bar: 100 μm. Data are presented as mean ± SEM. Statistics were acquired by two‐tailed unpaired Student's *t* test in A, B, D, E and F. *p < 0.05; **p < 0.01; ****p < 0.0001; n.s.: Not significant.

As described above, combinatorial treatment with Enza was capable of restoring sensitivity to PTX in resistant cells in vitro. We next sought to examine whether PTX and Enza in combination also displayed a synergistic effect in vivo. To this end, we established a xenograft mouse model using SUM159‐R cells, followed by either single or dual treatments. Consistent with in vitro data, neither single treatment affected tumour growth, whereas dual treatment led to a significant decrease in both tumour volume and weight (Figure 6A,B). In contrast, when AURKB was depleted by shRNA, the addition of Enza could not resensitise resistant cells to PTX anymore (Figure 6C,D). The effective inhibition of PKC by Enza was confirmed by immunohistochemical staining of phosphorylated GSK3β, a downstream marker indicating the activity of PKC (Figure S7). In line with the suppression of tumour growth, we also observed a significant decrease in the proliferation marker, Ki‐67, and an increase in the apoptotic marker, cleaved caspase3 in the combination group compared to either single treatment (Figure 6E,F). Collectively, these data demonstrated that the combination of PTX and Enza is a potent strategy to overcome paclitaxel resistance in TNBC in vivo, which largely depends on the accumulation of AURKB.

## 4 Discussion

Chemotherapy, like paclitaxel, remains a largely opted treatment approach for TNBC patients. Although some early‐stage patients could benefit from this conventional treatment, the overall prognosis is still poor due to resistance. Therefore, novel combination strategies are in dire need to improve the clinical efficacy of chemotherapy. Here, by performing drug screening targeting various kinases, we have identified that enzastaurin, which targets PKC, was able to reverse paclitaxel resistance in TNBC, both in vitro and in vivo.

The Protein Kinase C family is a group of Ser/Thr kinases and is involved in a wide variety of signalling transduction pathways that control cell proliferation, migration and apoptosis, thus being regarded as an attractive target for cancer treatment [49, 50]. Through analysis of diverse PKC isoforms at the RNA level, we found that PRKCA and PRKCD were the most abundant isoforms expressed in TNBC cell lines, which could be targeted by enzastaurin. Targeting PKCα/δ was able to restore sensitivity to paclitaxel in resistant TNBC cells. In line with our finding, the AXL‐PYK2‐PKCα circuit has been shown to regulate cancer cell stemness in TNBC, which is also considered an important root of therapeutic resistance and poor prognosis [24]. One of the canonical ways that paclitaxel executes its tumour cell killing function is to arrest cells in mitosis and simultaneously activate proapoptotic signalling pathways which ultimately culminates in cell death. In this study, we demonstrated that PKC inhibition was capable of strongly restoring mitotic arrest induced by paclitaxel, in turn leading to robust cell death in resistant cells. In addition to cell‐intrinsic mechanisms underlying resistance, accumulating evidence revealed that the tumour microenvironment could also significantly influence drug response and clinical outcomes, which can be mediated by ‘intermediate messengers’ such as cytokines, chemokines and exosomes [51, 52, 53]. Very interestingly, a recent seminal study demonstrated that the PKCα‐ZFP64‐CSF1 axis drives an immunosuppressive microenvironment and that inhibition of PKCα is capable of enhancing the efficacy of anti‐PD‐1 treatment via reshaping the tumour immune microenvironment [54]. These data suggested that targeting PKC might be a powerful strategy to overcome therapeutic resistance not only through a cell‐intrinsic manner but also by resetting the tumour microenvironment, which warrants further investigation in TNBC.

Mechanistically, the addition of enzastaurin could restore AURKB expression posttranscriptionally, therefore leading to efficient mitotic arrest as well as cell death. AURKB, as a member of the chromosome passenger complex, is able to phosphorylate chromatin proteins such as histone 3 to aid in mitotic chromosome condensation and is often overexpressed in a wide range of human cancers, including breast cancer. Although the role of AURKB as a cancer‐promoting gene has been widely studied, its role in regulating therapeutic resistance remains largely unknown [40, 42]. In the current study, we have uncovered that AURKB can modulate the response to the newly identified combinatorial treatment strategy in TNBC. Silencing of AURKB expression or pharmaceutical inhibition of its activity showed decreased sensitivity to paclitaxel/enzastaurin dual treatment, both in vitro and in vivo. Moreover, the expression of AURKB has previously been reported to be finely regulated primarily through a proteasome‐dependent manner during cell cycle control, whereas our study demonstrated a novel mechanism leading to AURKB accumulation via the GCN2‐p‐eIF2α axis upon combinatorial treatment. Intriguingly, this is highly consistent with another study, which documented the essential role of the GCN2‐p‐eIF2α axis in modulating response to inhibitors targeting oncogenic kinases, implying that activation of the GCN2‐p‐eIF2α signalling could be a promising way to conquer drug resistance [55].

**FIGURE 7:**
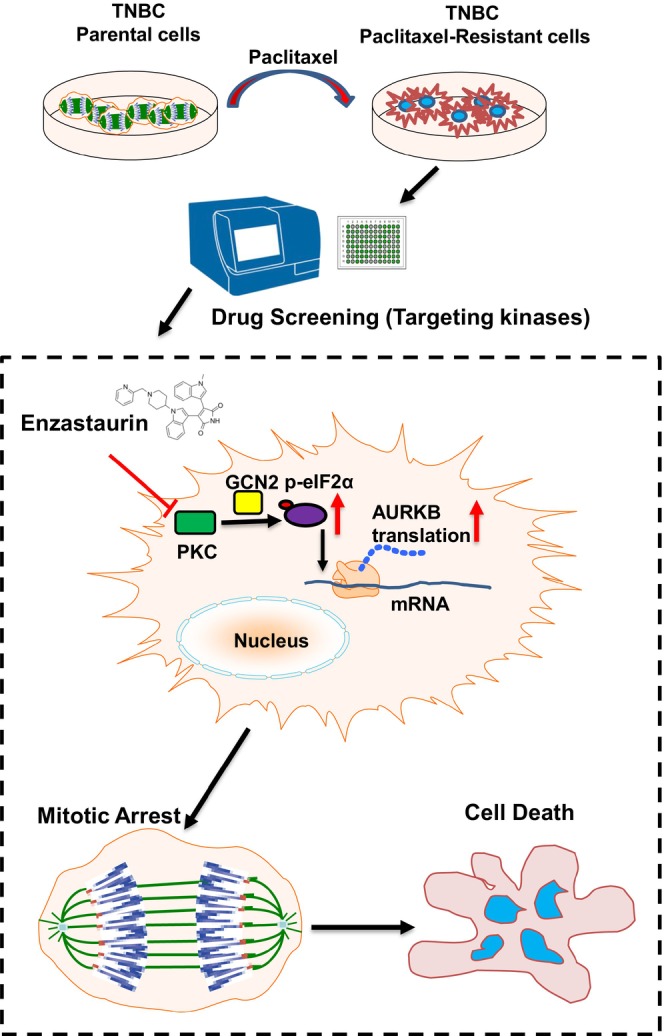
The proposed model of resensitising resistant TNBC to paclitaxel by targeting PKC. Schematic diagram depicting the underlying mechanism to override resistance of TNBC to paclitaxel by targeting PKC.

In summary, our study has illustrated a novel combinatorial treatment strategy to combat chemotherapeutic resistance in TNBC by restoring the expression of AURKB, which could be a potential predictive marker to stratify patients with paclitaxel‐resistant TNBC but who may benefit from the newly identified combination approach (Figure 7). Furthermore, as a potent pan‐inhibitor targeting PKCs, enzastaurin has entered clinical trials for the treatment of various cancers and has been granted fast‐track designation for the treatment of GBM by the FDA in 2020. This may rapidly advance the combination regimen into clinical trials for those TNBC patients encountering resistance to paclitaxel.

## Author Contributions

**Bing Cheng:** conceptualization (equal), data curation (equal), formal analysis (equal), funding acquisition (equal), investigation (equal), methodology (equal), project administration (equal), resources (equal), software (equal), supervision (equal), validation (equal), visualization (equal), writing – original draft (equal), writing – review and editing (equal). **Jinxin Chen:** data curation (equal), investigation (equal), methodology (equal), validation (equal), visualization (equal). **Vera Katalina:** data curation (equal), investigation (equal), methodology (equal), resources (equal), visualization (equal), writing – original draft (equal). **Guojie Long:** data curation (equal), investigation (equal), methodology (equal), validation (equal). **Chaoying Wei:** data curation (equal), methodology (equal), validation (equal). **Zhitong Niu:** data curation (equal), methodology (equal), software (equal), visualization (equal). **Chen Chen:** data curation (equal), methodology (equal), validation (equal). **Panpan Wang:** funding acquisition (equal), project administration (equal), resources (equal). **Qiang Yu:** conceptualization (equal), data curation (equal), funding acquisition (equal), project administration (equal), resources (equal), supervision (equal), writing – review and editing (equal). **Wenyu Wang:** conceptualization (equal), data curation (equal), formal analysis (equal), funding acquisition (equal), investigation (equal), methodology (equal), project administration (equal), resources (equal), software (equal), supervision (equal), validation (equal), visualization (equal), writing – original draft (equal), writing – review and editing (equal).

## Ethics Statement

The animal experiment was approved by the Animal Research Committee of the Sixth Affiliated Hospital of Sun Yat‐sen University (IACUC‐2022011201).

## Conflicts of Interest

The authors declare no conflicts of interest.

## Supporting information


**Data S1.** Additional materials and methods.

Data S2.
**Figures S1–S7**.

Data S3.
**Tables S1–S4**.

## Data Availability

Data available on request from the authors.
