# Supplementary material for: Targeting PKC as a Therapeutic Strategy to Overcome Chemoresistance in TNBC by Restoring Aurora Kinase B Expression
Source: J Cell Mol Med. 2025 Mar 18;29(6):e70464. doi: 10.1111/jcmm.70464 (PMC11915661; doi:10.1111/jcmm.70464)
Supplement: Supplementary file 1 — Data S1. Additional materials and methods. [file JCMM-29-e70464-s002.pdf]

Supporting Information

Supplementary Figure 1 Inhibitors targeting PKC don’t impair growth of parental TNBC cell lines.

Supplementary Figure 2 Enzastaurin shows minimal effect on sensitization of PTX resistant TNBC cell lines to Adriamycin.

Supplementary Figure 3 Combinatorial treatment with enzastaurin induces mitotic arrest in resistant TNBC cells.

Supplementary Fig 4 Silencing PRKCA/PRKCD induces mitotic arrest upon PTX treatment in resistant cell lines.

Supplementary Fig 5 Pharmaceutical inhibition of PKCα/δ by Go6983 promotes mitotic arrest in PTX resistant TNBC cell lines.

Supplementary Fig 6 Dual compound treatment shows modest effect on protein stability of AURKB compared to PTX alone in resistant cells.

Supplementary Fig 7 Enzastaurin shows effective inhibition of PKC activity indicated by phosphorylation of GSK3β at serine 9 in vivo.

Supplementary Tabe1 Antibodies used in this study

Supplementary Tabe2 Chemicals used in this study

Supplementary Table 3 SiRNAs and shRNAs used in this study

Supplementary Table 4 Primers used in this study

Additional materials and methods

Tumorsphere formation assay

Active growing cells were treated with 0.05% trypsin for 5 min then passed through 0.4 mm cell strainer, to achieve single cell suspension. Cells were plated in 6-well ultra-low attachment plate and cultured in MammoCultTM Basal Medium, supplemented with 10ml MammoCultTM Proliferation Supplements (Human) (STEMCELLTM Technologies, Vancouver, CA), 200µl heparin solution (stock 0.2%), and 200µl hydrocortisone (stock 250µg/ml). Tumor cells were cultured for 7 days and the number of tumor spheres with diameter>0.1 was considered for further analysis.

Anchorage-independent colony formation assay

The wells of an opaque 96-well plate with clear bottom were coated with Bacto™ Agar (#214010, BD Biosciences). The agar was first dissolved in PBS to make a 3% solution and boiled until dissolved. It was diluted with complete medium to make a 0.6% solution and 80µl were used to coat the bottom of each well. Plates were kept at 37°C, 5% CO2 for at least 45 mins before use. Methylcellulose powder (4,000 cP viscosity, 2% in H2O, Sigma Aldrich, St. Louis, MO) was autoclaved and dissolved in autoclaved water to make a 2% stock solution. Cells were resuspended in fresh medium and diluted to a final concentration of 2x104 cells/ml. 30ml of the cell solution were mixed together with 10ml of the 2% Methylcellulose stock solution. 1ml aliquots were made to which the drugs were added and then 100µl were seeded into each well onto the agar. The cells were grown for 13 days after which they were stained with 80µl of a 40µg/ml p-Iodonitortetrazolium Violet (INT) solution overnight. The colonies were

counted and analyzed using the GelCount™ automatic plate scanner (Oxford OPTRONIX) and the GelCount Version 0.025.1 software.

Flow cytometry analysis

Cells were fixed in 70% ice-cold ethanol for 1 hour, followed by treatment with RNase for 5 mins and stained with 50μg/ml Propidium Iodide (PI) solution for 30 mins at RT. The stained cells were analyzed for their DNA content by flow cytometry defined as the SubG1 population. For detection of apoptotic cells, cells were stained with the FITC-conjugated anti-active Caspase 3 antibody (BD Biosciences, #559341) according to the manufacturer's instructions. For mitosis analysis, cells were fixed in ice-cold 70% ethanol overnight, followed by incubation with anti-Phospho-Histone H3 (Ser28) antibody for 45 mins at RT. After that, cells were treated with RNase for 5 mins, followed by staining with PI for 30 mins. The percentage of mitotic cells were analyzed using flow cytometry.

RNA sequencing and data analysis

Total RNA was extracted and transcribed to cDNA with random primers. The cDNA was ligated to Illumina sequencing adaptors and sequenced on the Illumina NovaSeq6000 by Gene Denovo Biotechnology Co. (Guangzhou, China). A log2 (fold change) >2 and a false discovery rate (FDR) < 0.01 were used as cut-off thresholds. Raw and normalized RNA sequencing data generated in this study was deposited into the gene expression omnibus (GEO) archive under the accession number GSE250221.
