# Supplementary material for: Targeting PKC as a Therapeutic Strategy to Overcome Chemoresistance in TNBC by Restoring Aurora Kinase B Expression
Source: J Cell Mol Med. 2025 Mar 18;29(6):e70464. doi: 10.1111/jcmm.70464 (PMC11915661; doi:10.1111/jcmm.70464)
Supplement: Supplementary file 2 — Data S2. Figures S1–S7. [file JCMM-29-e70464-s003.pdf]

Supplementary Fig 1 Inhibitors targeting PKC don't impair growth of parental TNBC cell lines

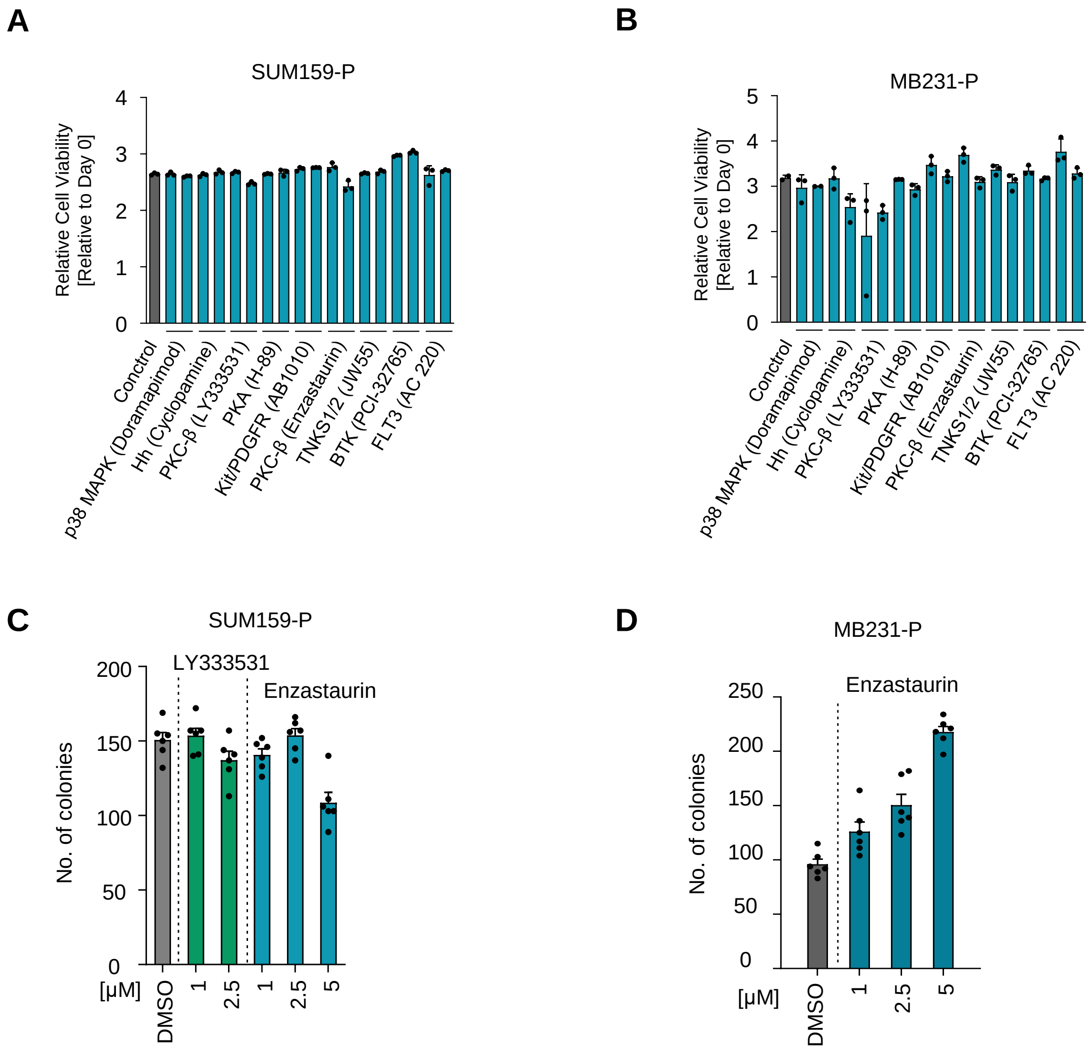

Supplementary Fig 1 Inhibitors targeting PKC doesn’t impair growth of parental TNBC cell lines

A, B. Measurement of cell viability upon treatment with inhibitors targeting candidate kinases in SUM159-P and MB231-P cells at dose of 1μM and 5μM.

C, D. Measurement of anchorage-independent cell growth upon treatment with inhibitors targeting PKC in SUM159-P and MB231-P cells at indicated doses.

Data are presented as mean ± SEM.

Supplementary Fig 2 Enzastaurin shows minimal effect on sensitization of PTX resistant TNBC cell lines to Adriamycin

A

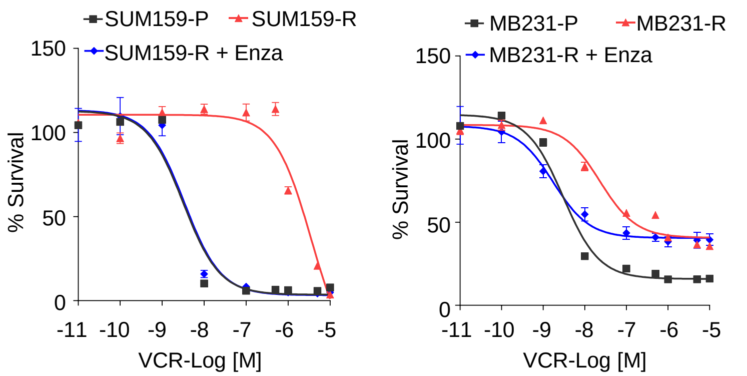

	EC50 (µM)
SUM159-P	3.17×10^{-6}
SUM159-R	3.61×10^{-3}
SUM159-R + Enza	3.38×10^{-6}

	EC50 (µM)
MB231-P	3.011×10^{-6}
MB231-R	2.321×10^{-5}
MB231-R + Enza	1.727×10^{-6}

B

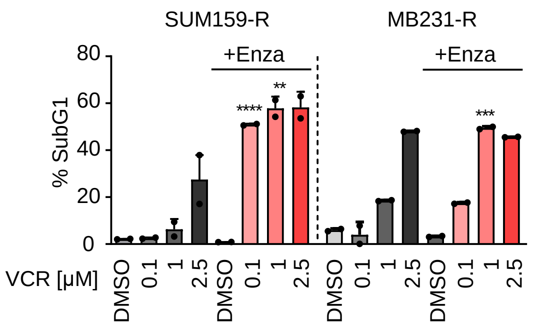

C

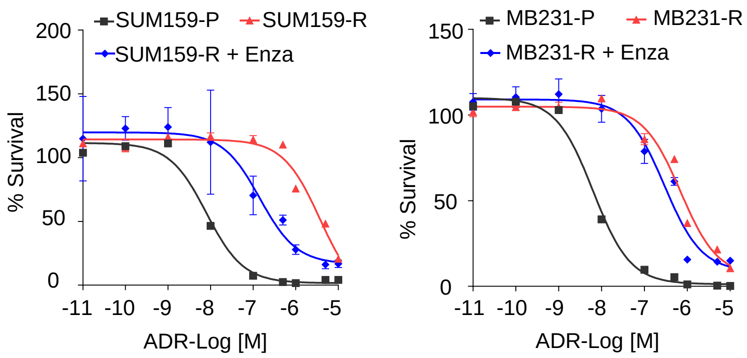

	EC50 (µM)
SUM159-P	7.993×10^{-6}
SUM159-R	3.724×10^{-3}
SUM159-R + Enza	1.430×10^{-4}

	EC50 (µM)
MB231-P	6.340×10^{-6}
MB231-R	6.979×10^{-4}
MB231-R + Enza	2.935×10^{-4}

D

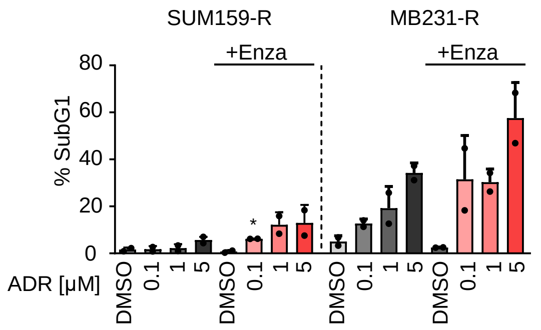

Supplementary Fig 2 Enzastaurin shows minimal effect on sensitization of PTX resistant TNBC cell lines to Adriamycin

A. Measurement of IC50 of both parental and resistant TNBC cell lines to the commonly used microtubule targeting drug-Vincristine(VDR) in combination with Enza or not.
B. FACS analysis of cell death stained with PI in both SUM159-R and MB231-R cells upon treatment with VDR alone or in combination with Enza at indicated doses.
C. Measurement of IC50 of parental and resistant TNBC cell lines to the commonly used anthracycline antibiotic drug-Adriamycin (ADR) in combination with Enza or not.
D. FACS analysis of cell death stained with PI in both SUM159-R and MB231-R cells upon treatment with ADR alone or in combination with Enza at indicated doses.

Data are presented as mean ± SEM. Statistics were acquired by two-tailed unpaired Student's t test in B. ***p*<0.01; ****p*<0.001; *****p*<0.0001; n.s.: not significant.

Supplementary Fig 3 Combinatorial treatment with enzastaurin induces mitotic arrest in resistant TNBC cells

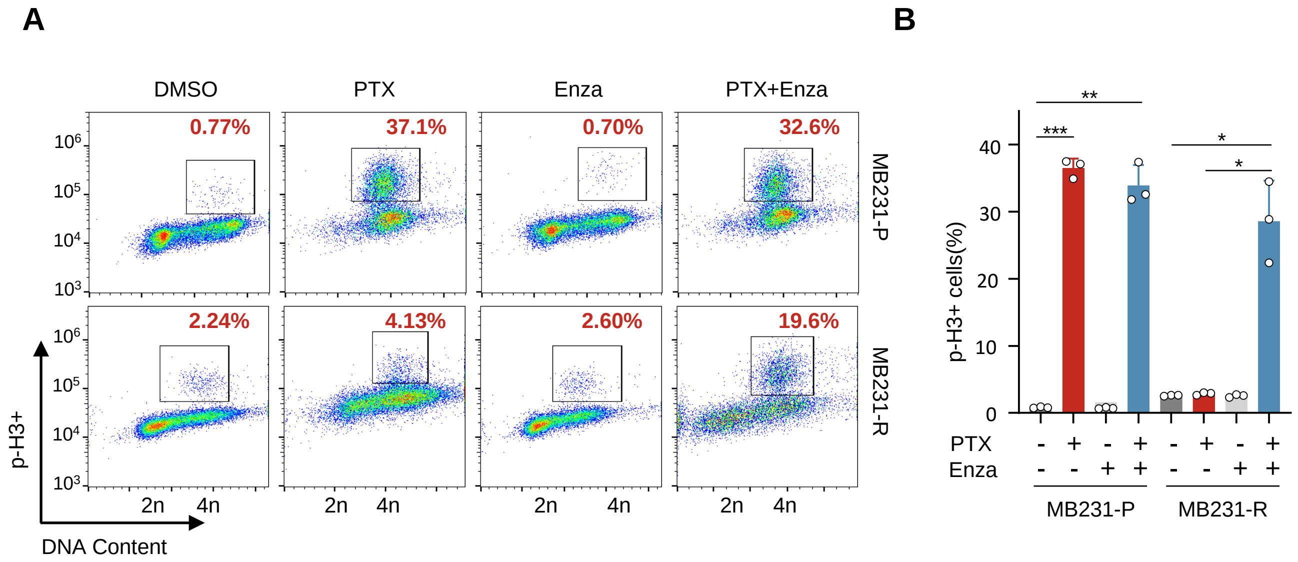

Supplementary Fig 3 Combinatorial treatment with enzastaurin induces mitotic arrest in resistant TNBC cells

A, B. FACS analysis of mitotic cells co-stained with phosphorylated Histone3 (p-H3, mitotic marker) and PI (DNA content) upon indicated treatment in both MB231-P and MB231-R cells. (A) Representative images from FACS analysis of mitotic cells. The red number represents proportion of mitotic cells under indicated treatment.(B) Statistical analysis of three independent experiments.

Data are presented as mean ± SEM. Statistics were acquired by two-tailed unpaired Student’s t test in B. *p<0.05; **p<0.01; ***p<0.001; n.s.: not significant.

Supplementary Fig 4 Silencing PRKCA/PRKCD induces mitotic arrest upon PTX treatment in resistant cell lines

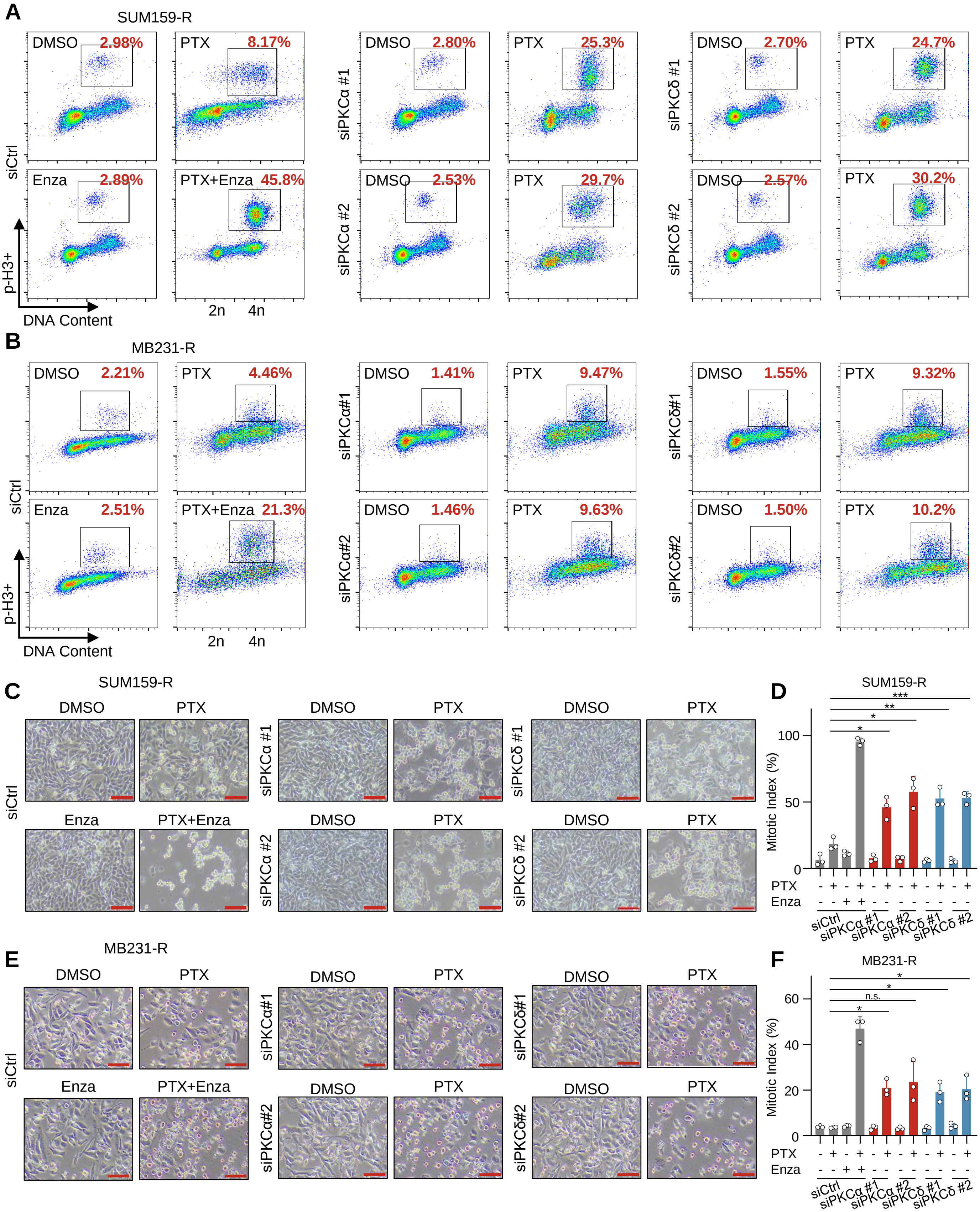

Supplementary Fig 4 Silencing PRKCA/PRKCD induces mitotic arrest upon PTX treatment in resistant cell lines

A, B. FACS analysis of mitotic cells co-stained with phosphorylated Histone3 (p-H3, mitotic marker) and PI (DNA content) following transfection with control siRNA or two independent siRNA targeting PKCα or PKCδ upon indicated treatment in both SUM159-R and MB231-R cells. Representative images from FACS analysis of mitotic cells in SUM159-R cells (A) and MB231-R cells (B). The red number represents proportion of mitotic cells under indicated treatment.

C, D. Representative images showing round-up mitotic cells following transfection with control siRNA or two independent siRNA targeting PKCα or PKCδ upon indicated treatment in SUM159-R cells. Scale bar: 100μM. Mitotic Index was calculated by measuring percentage of mitotic cells among total cells in the same field. 5-8 fields were counted per condition. The plot showing statistical analysis from three independent experiments.

E, F. Representative images showing mitotic cells following transfection with control siRNA or two independent siRNA targeting PKCα or PKCδ upon indicated treatment in MB231-R cells. Scale bar: 100μM. The plot showing statistical analysis from three independent experiments.

Data are presented as mean ± SEM. Statistics were acquired by two-tailed unpaired Student's t test in D and E. *p<0.05; **p<0.01; ***p<0.001; n.s.: not significant.

Supplementary Fig 5 Pharmaceutical inhibition of PKCα/δ by Go6983 promotes mitotic arrest in PTX resistant TNBC cell lines

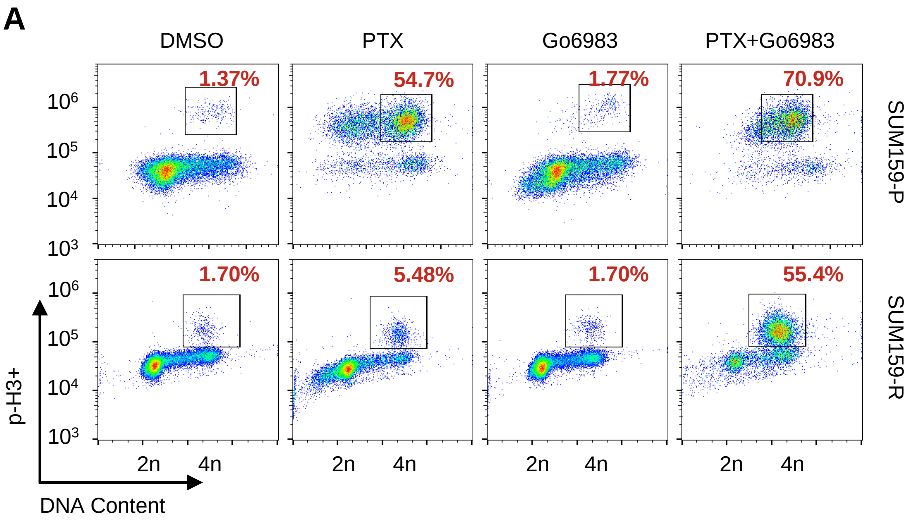

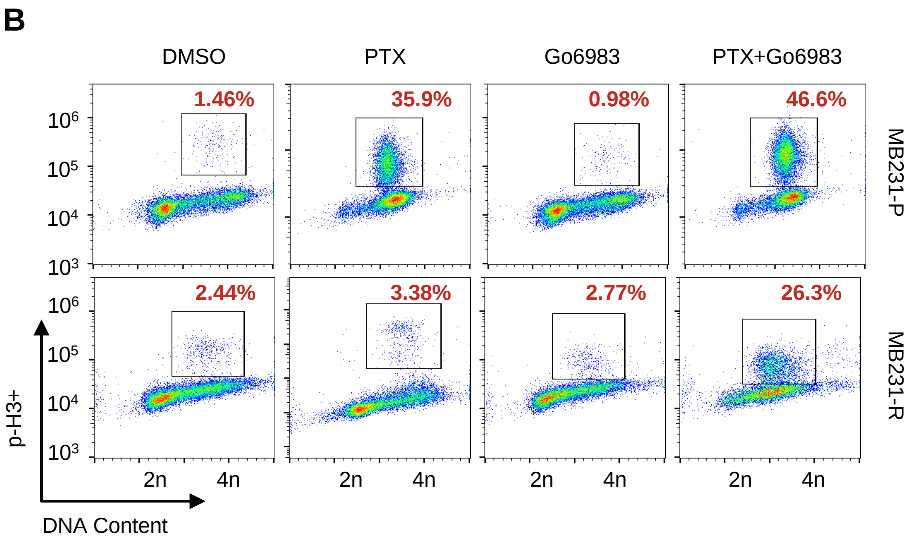

Supplementary Fig 5 Pharmaceutical inhibition of PKCα/δ by Go6983 promotes mitotic arrest in PTX resistant TNBC cell lines

A. Representative images showing FACS analysis of mitotic cells co-stained with p-H3 and PI upon treatment with either PTX / PKC inhibitor (Go6983) alone or in combination in SUM159-P cells and SUM159-R cells. The red number represents proportion of mitotic cells under indicated treatment.

B. Representative images showing FACS analysis of mitotic cells co-stained with p-H3 and PI upon treatment with either PTX / PKC inhibitor (Go6983) alone or in combination in MB231-P cells and MB231-R cells.

Supplementary Fig 6 Dual compound treatment shows modest effect on protein stability of AURKB compared to PTX alone in resistant cells

A

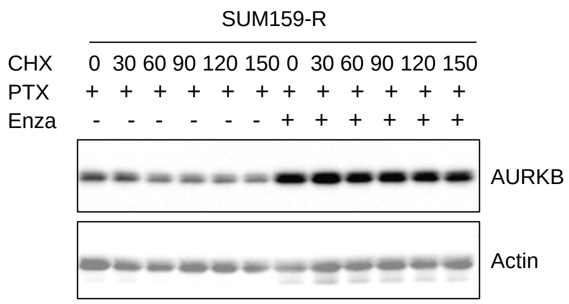

B

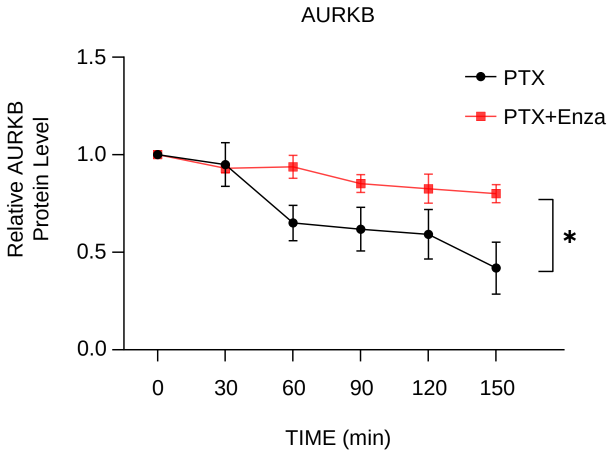

Supplementary Fig 6 Dual compound treatment shows modest effect on protein stability of AURKB compared to PTX single treatment in resistant cells

A. Western blot analysis of AURKB expression in SUM159-R cells upon treatment with PTX alone or in combination with Enza in the presence of cycloheximide (CHX; 200μM) for the indicated time points.

B. The plot showing quantification of relative protein level of AURKB treated as described in (A) from three independent experiments.

All immunoblot analysis was representative of three independent experiments. Data are presented as mean ± SEM. Statistics were acquired by two-tailed unpaired Student's t test in B. *p<0.05.

Supplementary Fig 7 Enzastaurin shows effective inhibition of PKC activity indicated by phosphorylation of GSK3β at serine 9 in vivo

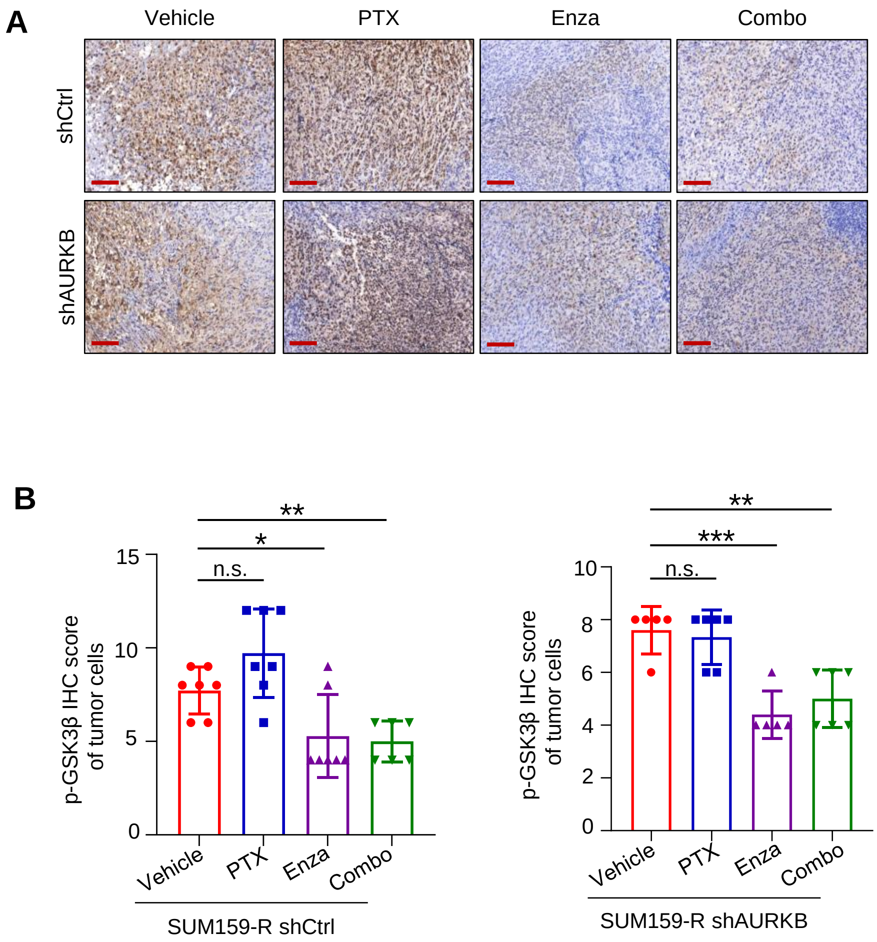

Supplementary Fig 7 Enzastaurin shows effective inhibition of PKC activity indicated by phosphorylation of GSK3β at serine 9 in vivo

A. Immunohistochemical staining of phosphorylated GSK3β at serine 9 in the tumor sections from shCtrl or shAURKB group upon treatment with PTX / Enza alone or in combination. Scale bar: 100μm.
B. The plot showing quantified IHC score of phosphorylated GSK3β at serine 9 in the tumor sections from shCtrl or shAURKB group upon treatment with PTX / Enza alone or in combination.

Data are presented as mean ± SEM. Statistics were acquired by two-tailed unpaired Student's t test in B. *p<0.05; **p<0.01; ***p<0.001; n.s.: not significant.
