# Supplementary material for: Targeting PKC as a Therapeutic Strategy to Overcome Chemoresistance in TNBC by Restoring Aurora Kinase B Expression
Source: J Cell Mol Med. 2025 Mar 18;29(6):e70464. doi: 10.1111/jcmm.70464 (PMC11915661; doi:10.1111/jcmm.70464)
Supplement: Supplementary file 3 — Data S3. Tables S1–S4. [file JCMM-29-e70464-s001.pdf]

Supplementary Table 1. Antibodies used in this study

Antibody	Cat.No and Company
**phospho-GSK3β (Ser9)**	#5558S, Cell Signaling Technology
**GSK3β**	#51065-1-AP, Proteintech
**Aurora B/AIM1**	#3094S, Cell Signaling Technology
**eIF2α**	#5324S, Cell Signaling Technology; #11233-1-AP, Proteintech
**p-eIF2α**	#3398S, Cell Signaling Technology
**GCN2**	#SC-374609, Santa Cruz
**p-GCN2(Thr899)**	#AF8154, Affinity
**Actin**	#A5441, Sigma
**Cleaved Caspase-3 (Asp175)**	#9661S, Cell Signaling Technology
**Phosphor-Histone H3**	#9713, Cell Signaling Technology
**Ki67**	#ab15580, Abcam

Supplementary Table 2. Chemicals used in this study

Chemical reagent	Cat. No and Company
**Paclitaxel**	HY-B0015, MedChemExpress
**Enzastaurin(PKC inhibitor)**	T6280, Tegretol
**Doramapimod (p38 MAPK inhibitor)**	1358, Axon Medchem
**LY333531(PKC-β inhibitor)**	1401, Axon Medchem
**AB1010 (Kit/PDGFR inhibitor)**	1419, Axon Medchem
**JW-55 (Tankyrase inhibitor)**	1922, Axon Medchem
**PCI-32765 (Bruton's Tyrosine kinase inhibitor)**	1858, Axon Medchem
**AC-220 (FLT3 inhibitor)**	1696, Axon Medchem

**Cyclosporine (Hedgehog inhibitor)**	C-8700, LC Labs
**H-89 (PKA inhibitor)**	H-5239, LC Labs
**AZD 1152-HQPA (Aurora kinase inhibitor)**	1580, Axon Medchem
**MLN8237(Aurora kinase inhibitor)**	2003, Axon Medchem
**ZM447439 (Aurora kinase inhibitor)**	1541, Axon Medchem
**Tak-901**	S2718, Selleck
**VX-680**	T2304, LC Labs
**Go-6983 (pan-PKC inhibitor)**	HY-13689, MedChemExpress
**GCN2iB (GCN2 inhibitor)**	HY-112654, MedChemExpress
**Sal003(eIF2α phosphatase inhibitor)**	HY-15969, MedChemExpress
**RSL3(GPX4 inhibitor)**	HY-100218A, MedChemExpress
**BODIPY™ 581/591 C11**	D3861, ThermoFisher Scientific

Supplementary Table 3. SiRNAs and shRNAs used in this study

SiRNA and shRNA	Sequence
**si AURKB**	5’-AACGCGGCACUUCACAAUUGA-3’
**si PKCα #1:**	5-CUUUGGAGUUUCGGAGCUGAT-3
**si PKCα#2:**	5-CCGAGUGAAACUCACGGACUUCAAU-3
**si PKCδ #1:**	5-GCAAGACAACAGUGGGACCUA-3
**si PKCδ #2:**	5-GGCCGCUUUGAACUCUACCGU-3
**siGCN2 #1:**	5-GGUCCAAGGAAGCACCAAA-3
**siGCN2 #2:**	5-CCAAAGGUCUAUCAAAUGAAA-3
**shAURKB**	5-AACGCGGCACTTCACAATTGA-3

Supplementary Table 4. Primers used in this study

Gene	Forward (5'-3')	Reverse (5'-3')
**MUC1**	CCTACCATCCTATGAGCGAGTAC	GCTGGGTTTGTGTAAGAGAGGC
**MUC15**	GGTGTCTCATTGCTTACTCTTGTG	CCAAAACTCACATCATAAGGTTCC
**CLDN4**	AGTGCAAGGTGTACGACTCGCT	CGCTTTCATCCTCCAGGCAGTT
**TWIST1**	AGTCCGCAGTCTTACGAGGA	GCCAGCTTGAGGGTCTGAAT
**TWIST2**	GCAAGATCCAGACGCTCAAGCT	ACACGGAGAAGGCGTAGCTGAG
**VIM**	ACTACGTCCACCCGCACCTA	CAGCGAGAAGTCCACCGAGT
**ZEB2**	AATGCACAGAGTGTGGCAAGGC	CTGCTGATGTGCGAACTGTAGG
**ACTA2**	GGTGACGAAGCACAGAGCAA	CAGGGTGGGATGCTCTTCAG
**EPCAM**	GCCAGTGTACTTCAGTTGGTGC	CCCTTCAGGTTTTGCTCTTCTCC
**SNAIL2**	ATCTGCGGCAAGGCGTTTTCCA	GAGCCCTCAGATTTGACCTGTC
**FN1**	ACAACACCGAGGTGACTGAGAC	GGACACAACGATGCTTCCTGAG
**NCAD**	TACACTGCCCAGGAGCCAGA	TGGCACCAGTGTCCGGATTA
**HMOX1**	AAGACTGCGTTCCTGCTCAAC	AAAGCCCTACAGCAACTGTCG
**SOD1**	CTCACTCTCAGGAGACCATTGC	CCACAAGCCAAACGACTTCCAG
**SOD2**	CTGGACAAACCTCAGCCCTAAC	AACCTGAGCCTTGGACACCAAC
**SOD3**	ATGCTGGCGCTACTGTGTTC	CTCCGCCGAGTCAGAGTTG
**CBS**	CATTGCCAGGAAGCTGAAGGAG	TTCCACCTCGTAGGTTGTCTGC
**CTH**	GGCCTGGTGTCTGTTAATTGT	GGCCTGGTGTCTGTTAATTGT
**FLT**	TACGAGCGTCTCCTGAAGATGC	GGTTCAGCTTTTTCTCCAGGGC
**PRKCA**	GTCCACAAGAGGTGCCATGAA	AAGGTGGGGCTTCCGTAAGT
**PRKCB**	AAACCTTGTACCTATGGACCCC	CCCAATCCCAAATCTCTACTGAC
**PRKCD**	TGTGCCGTGAAGATGAAGGAG	TAGATGTGGGCATCGAACGTC
**PRKCE**	CAGGGATTTGAAACTGGACA	CTGCAGGATCTCAGGAGCTA

**PRKCG**	CACGAAGTCAAGAGCCACA	TAGCTATGCAGGCGGAACTT
**Actin**	CACCATTGGCAATGAGCGGTTC	AGGTCTTTGCGGATGTCCACGT
